# Safety and Glycemic Outcomes During the MiniMed™ Advanced Hybrid Closed-Loop System Pivotal Trial in Adolescents and Adults with Type 1 Diabetes

**DOI:** 10.1089/dia.2021.0319

**Published:** 2022-03-14

**Authors:** Anders L. Carlson, Jennifer L. Sherr, Dorothy I. Shulman, Satish K. Garg, Rodica Pop-Busui, Bruce W. Bode, David R. Lilenquist, Ron L. Brazg, Kevin B. Kaiserman, Mark S. Kipnes, James R. Thrasher, John H. Chip Reed, Robert H. Slover, Athena Philis-Tsimikas, Mark Christiansen, Benyamin Grosman, Anirban Roy, Melissa Vella, Richard A.M. Jonkers, Xiaoxiao Chen, John Shin, Toni L. Cordero, Scott W. Lee, Andrew S. Rhinehart, Robert A. Vigersky

**Affiliations:** ^1^ International Diabetes Center, HealthPartners Institute, Minneapolis, Minnesota, USA.; ^2^ Yale University School of Medicine Pediatric Endocrinology, New Haven, Connecticut, USA.; ^3^ University of South Florida Diabetes and Endocrinology, Tampa, Florida, USA.; ^4^ Barbara Davis Center of Childhood Diabetes, Aurora, Colorado, USA.; ^5^ Division of Metabolism, Endocrinology and Diabetes, University of Michigan, Ann Arbor, Michigan, USA.; ^6^ Atlanta Diabetes Associates, Atlanta, Georgia, USA.; ^7^ Rocky Mountain Diabetes and Osteoporosis Center, Idaho Falls, Idaho, USA.; ^8^ Rainier Clinical Research Center, Renton, Washington, USA.; ^9^ SoCal Diabetes, Torrance, California, USA.; ^10^ Diabetes and Glandular Disease Clinic, San Antonio, Texas, USA.; ^11^ Arkansas Diabetes and Endocrinology Center, Little Rock, Arkansas, USA.; ^12^ Endocrine Research Solutions, Inc., Roswell, Georgia, USA.; ^13^ Scripps Whittier Diabetes Institute, La Jolla, California, USA.; ^14^ Diablo Clinical Research Center, Walnut Creek, California, USA.; ^15^ Medtronic, Northridge, California, USA.

**Keywords:** Type 1 diabetes, A1C, Time-in-range, Advanced hybrid closed loop, Adolescents, Adults

## Abstract

**Introduction:**

This trial assessed safety and effectiveness of an advanced hybrid closed-loop (AHCL) system with automated basal (Auto Basal) and automated bolus correction (Auto Correction) in adolescents and adults with type 1 diabetes (T1D).

**Materials and Methods:**

This multicenter single-arm study involved an intent-to-treat population of 157 individuals (39 adolescents aged 14–21 years and 118 adults aged ≥22–75 years) with T1D. Study participants used the MiniMed™ AHCL system during a baseline run-in period in which sensor-augmented pump +/− predictive low glucose management or Auto Basal was enabled for ∼14 days. Thereafter, Auto Basal and Auto Correction were enabled for a study phase (∼90 days), with glucose target set to 100 or 120 mg/dL for ∼45 days, followed by the other target for ∼45 days. Study endpoints included safety events and change in mean A1C, time in range (TIR, 70–180 mg/dL) and time below range (TBR, <70 mg/dL). Run-in and study phase values were compared using Wilcoxon signed-rank test or paired *t*-test.

**Results:**

Overall group time spent in closed loop averaged 94.9% ± 5.4% and involved only 1.2 ± 0.8 exits per week. Compared with run-in, AHCL reduced A1C from 7.5% ± 0.8% to 7.0% ± 0.5% (<0.001, Wilcoxon signed-rank test, *n* = 155), TIR increased from 68.8% ± 10.5% to 74.5% ± 6.9% (<0.001, Wilcoxon signed-rank test), and TBR reduced from 3.3% ± 2.9% to 2.3% ± 1.7% (<0.001, Wilcoxon signed-rank test). Similar benefits to glycemia were observed for each age group and were more pronounced for the nighttime (12 AM–6 AM). The 100 mg/dL target increased TIR to 75.4% (*n* = 155), which was further optimized at a lower active insulin time (AIT) setting (i.e., 2 h), without increasing TBR. There were no severe hypoglycemic or diabetic ketoacidosis events during the study phase.

**Conclusions:**

These findings show that the MiniMed AHCL system is safe and allows for achievement of recommended glycemic targets in adolescents and adults with T1D. Adjustments in target and AIT settings may further optimize glycemia and improve user experience.

Clinical Trial Registration number: NCT03959423.

## Introduction

Iterative advances in automated (closed loop) insulin delivery have provided clinically significant improvements in glycemia, with the ultimate goal of simultaneously reducing the burden of diabetes management. Early trials investigating closed-loop algorithms reported overall safety and improvements in 24-h day and overnight time spent in the target sensor glucose (SG) range of 70–180 mg/dL (TIR), hemoglobin A1C, and/or mean SG, without increased exposure to hypoglycemia, when compared with open-loop control.^1–8^ Prospective closed-loop studies have also demonstrated increased user satisfaction and/or reduced diabetes-related burden.^9–11^ Nevertheless, many individuals living with diabetes have yet to achieve the American Diabetes Association glycemic targets.^12,13^

Although A1C has represented the clinical gold standard average of blood glucose, this metric's inability to capture the frequency, duration, and severity of hypoglycemia and hyperglycemia like continuous glucose monitoring (CGM) data limits its utility in day-to-day and within-day treatment decisions. The report and use of CGM data in clinical studies and patient management, through either a standalone device or integrated component in automated insulin delivery devices, have led to newly established goals for glycemia. The International Consensus on Use of Continuous Glucose Monitoring recommended standards for reporting CGM metrics^14^ and recommended goals for time spent across SG ranges.^15^ Recommended percentages of time spent within 70–180 mg/dL, >180 mg/dL, and <70 mg/dL ranges (TIR, TAR, and TBR, respectively) have evolved to become standard metrics that supplement A1C.

The pivotal trials of the first Food and Drug Administration-approved hybrid closed-loop therapy, the Medtronic MiniMed™ 670G system, demonstrated an increase in 24-h day and nighttime TIR, a reduction in TBR and TAR, and a lowered mean A1C when compared with open-loop use, in children,^16^ adolescents, and adults.^17^ The median time spent in Auto Mode for each group was 80.6%, 75.8%, and 88.0%, respectively. For each trial's Auto Mode-enabled study phase, there were no episodes of severe hypoglycemia or diabetic ketoacidosis (DKA). Real-world MiniMed 670G system analyses have validated the aforementioned pivotal trial findings with respect to TIR and reduction in both TBR and TAR in individuals living in the United States^18^ and 13 different European countries.^19^

Although the extended use of MiniMed 670G Auto Mode has been shown to significantly improve real-world glycemia,^20–22^ challenges with adherence to system therapy have been reported. Frequent calibration requests and system Auto Mode exits have been reasons cited for discontinuing system use.^23–25^ Consequently, a next-generation therapy was designed with an algorithm that reduces closed-loop exits and provides automated bolus insulin corrections every 5 min, in addition to the existing automated basal insulin delivery and low glucose management features of the MiniMed 670G system.

This advanced hybrid closed-loop (AHCL) system includes a meal detection module developed by DreaMed Diabetes (Petah Tikvah, Israel) that, if triggered, can allow for a more aggressive auto-correction bolus when appropriate. In addition, the AHCL system provides the option of setting two different glucose targets, 100 or 120 mg/dL, as well as the temporary target of 150 mg/dL.

There have been several small- and short-term randomized controlled trials (RCTs) of the AHCL system in adolescents in a camp setting,^26^ adolescents and young adults in a supervised followed by at-home setting,^27^ and adults in a supervised live-in setting^28^ that demonstrated improved glycemia. Furthermore, these trials highlighted a significant duration of time spent in closed loop (>90%), with each reporting reduced or minimal exits. The RCTs capturing participant-reported outcomes also observed improved user satisfaction with the AHCL system, when compared with MiniMed 670G HCL system use.^26,28^ These improvements in glycemia, time in closed-loop control, and user satisfaction trended similarly with those observed in a different insulin delivery system (Tandem^®^ Diabetes Care, San Diego, CA) with automated basal and bolus functions.^29^

More recently, larger and longer duration (4–12 weeks per intervention and control) AHCL system RCTs in youth and adults,^30,31^ in addition to a brief longitudinal AHCL system evaluation,^32^ have reported time in closed loop >90% and demonstrated significantly improved glycemia in endpoints that include overall TIR, TAR, and mean SG, while reducing or not changing time spent in hypoglycemia. In addition, they have also shown that specific AHCL system settings (i.e., glucose target and active insulin time [AIT]) can optimize overall TIR.^31,32^

This study assessed safety and change in glycemia (i.e., A1C, TIR, and other descriptive endpoints) in adolescents and adults with type 1 diabetes (T1D) during the Medtronic *Safety Evaluation of the Advanced Hybrid Closed Loop (AHCL) System in Type 1 Adult and Pediatric Subjects* trial.

## Methods

### Study design

**FIG. 1.**
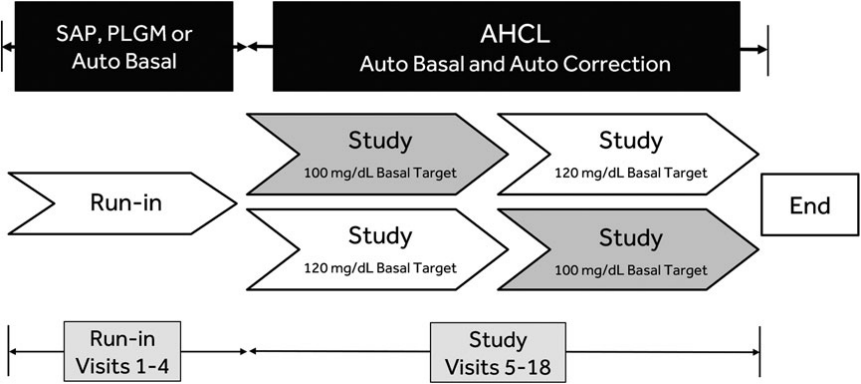
Study flow. The study schedule included a run-in period (Visits 1–4) with SAP with or without predictive low glucose management or automated basal (Auto Basal) use for ∼14 days, and a study phase (Visits 5–18) with Auto Basal and automated bolus correction (Auto Correction) enabled for ∼90 days. A glucose target of 100 or 120 mg/dL was set during the first 45 ± 5 days of the study phase, which was followed by a switch to the other glucose target for the remaining 45 ± 5 days. AHCL, advanced hybrid closed loop; PLGM, predictive low glucose management; SAP, sensor-augmented/integrated pump.

This multicenter open-label single-arm study enrolled adolescents (14–21 years of age) and adults (22–75 years of age) with T1D. Participants who met inclusion and exclusion criteria underwent consent and screening before entry into a baseline run-in period comprising Visits 1–4, followed by a study phase comprising Visits 5–18 (Fig. 1). Both the run-in period and study phase involved use of the AHCL study device that included the MiniMed 670G insulin pump (version 4.0 algorithm) with CGM system (the Guardian™ Sensor [3] glucose sensor and Guardian Link [3] transmitter).

During the run-in period, Visits 1–3 included study eligibility confirmation, pump training and initiation, and CGM training, with measured sensor wear (∼14 days) beginning at Visit 3. Participants were able to use the system as a sensor-augmented pump (SAP) with or without predictive low glucose management (PLGM) or closed-loop therapy with automated basal delivery (Auto Basal), but not automated insulin correction bolus (Auto Correction), until study phase start. At the beginning of the study phase (Visit 5), participants were instructed to enable both Auto Basal and Auto Correction with either a 100 or 120 mg/dL glucose target for ∼45 days. Thereafter, the other target was to be programmed for the remaining ∼45 days of the study phase.

Institutional review board approval was obtained for each investigational center. Informed consent or assent was obtained in accordance with the Code of Federal Regulations (CFR) Title 21, Part 50. Medical oversight during the study involved investigational center staff with appropriate medical training and a physician (principal investigator) or designee who has managed persons with diabetes using both CGM and insulin pump therapy.

During the run-in period and study phase, the incidence of each of the following safety events was captured: serious adverse events (SAEs), serious adverse device effects (SADEs), unanticipated adverse device effects, severe hypoglycemia, and DKA.

### Inclusion/exclusion criteria

General study inclusion criteria included a diagnosis of T1D at screening and a duration of T1D for, at least, 2 years. Additional key inclusion criteria included the participant having a minimum daily insulin requirement of, at least, 8 U; a hemoglobin A1C of <10% at screening; use of pump therapy with or without CGM experience for >6 months before screening; willingness to wear the system throughout the study and perform at least four daily self-monitoring of blood glucose measurements and required sensor calibrations; an ability to upload data from the study device to the CareLink™ clinical software; and a caregiver available at night who resided (or lived) in the same building (or home), during the study.

Individuals were excluded from taking part in the study for any one of the following: a history of one or more episodes of severe hypoglycemia that resulted in a coma, seizure, or hospitalization during the 6 months before screening; hospitalization or emergency room visit resulting in a primary diagnosis of uncontrolled diabetes in the 6 months before screening; DKA in the 6 months before screening; hypoglycemia unawareness, as measured by the Gold questionnaire^33^ at screening; or an inability to tolerate tape adhesive in the area of sensor placement or an unresolved adverse skin condition (e.g., psoriasis, dermatitis herpetiformis, rash, or staphylococcus infection) in the area of sensor placement.

### Statistical analyses

A post hoc sensitivity analysis conducted on the endpoints of the intent-to-treat (ITT) population of 157 participants entering the study phase and the per-protocol population of 132 participants confirmed equivalent robustness. As such, analyses conducted on changes in outcomes or descriptive comparisons from the run-in period to the end of the study phase were based on the ITT population.

Analyses of primary and secondary efficacy endpoints were exploratory. The study endpoints were the overall change in the mean TIR (70–180 mg/dL) and mean A1C from the end of the run-in period to the end of the study phase. Analyses of additional and/or descriptive endpoints included change in mean SG, coefficient of variation (CV) of SG, percentage of time spent at hypoglycemic ranges (<50, 54, and 70 mg/dL), percentage of time spent at hyperglycemic ranges (>180, 250, and 300 mg/dL), total daily dose of insulin (TDD) or total insulin per period, total basal [basal+microbolus] insulin, total bolus insulin, and Auto Correction in units, as well as a percentage of total bolus insulin. Subgroup analyses based on glucose target setting and AIT setting and the number of closed loop exits were also determined. The daytime period was defined as 6 AM–12 AM and the nighttime period was defined as 12 AM–6 AM.

For all comparisons, values were averaged per participant and compared between the run-in period and study phase using a Wilcoxon signed-rank test or paired *t*-test. Univariate associations between AIT setting and TIR and TBR were determined using a Spearman's rank-order correlation. Analyses were performed using SAS^®^ 9.4 (SAS Institute, Cary, NC).

## Results

### Study participant disposition

**FIG. 2.**
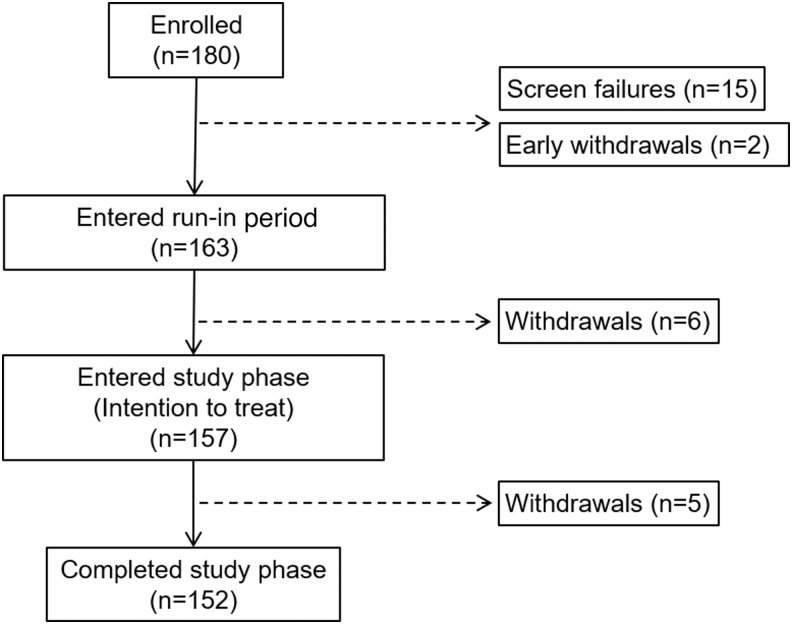
Participant disposition.

The study enrolled 180 individuals aged 14–75 years. A total of 163 participants entered the run-in period, 157 participants entered the study phase, and 152 completed the study (Fig. 2). The ITT population of 157 (mean of 38.3 ± 17.6 years of age) included 39 adolescents (16.2 ± 2.1 years) and 118 adults (45.6 ± 14.0 years). The overall group mean ± standard deviation of A1C at run-in was 7.5% ± 0.8% and ranged from 5.7% to 9.8%. Group demographics, therapy used, and other characteristics at baseline, in addition to those of the adolescent and adult groups, are given in Table 1.

**Table 1.** Demographic and Baseline Characteristics

	Overall (*n* = 157)	Adolescents (*n* = 39)	Adults (*n* = 118)
Age, years	38.3 ± 17.6	16.2 ± 2.1	45.6 ± 14.0
Female, *n* (%)	86 (54.8)	23 (59.0)	63 (53.4)
A1C, %	7.5 ± 0.8	7.6 ± 0.8	7.5 ± 0.9
Diabetes duration, years	22.6 ± 13.3	9.2 ± 3.7	27.0 ± 12.3
Weight, kg	80.1 ± 18.5	68.8 ± 11.9	83.9 ± 18.8
BMI, kg/m^2^	27.5 ± 5.7	24.2 ± 4.0	28.6 ± 5.8
Therapy			
HCL	82	25	57
SAP	70	13	57
CSII	5	1	4

All data are shown as means ± SD, excluding gender.

BMI, body mass index; CSII, continuous subcutaneous insulin infusion; HCL, hybrid closed loop; SAP, sensor-augmented pump; SD, standard deviation.

### Safety events

Throughout this study of >20,229 days, there were three SAEs (one severe hypoglycemia in an adolescent during the run-in period and one appendicitis and one sepsis secondary to pyelonephritis during the study phase), all of which were not related to the investigational device. There were no episodes of DKA, SADEs, or unanticipated device effects.

### System usability and impact on glycemia

For all participants, >94% of the time was spent in closed loop and, based on the 24-h day, there were 1.2 ± 0.8 exits per participant per week. The most common reasons for exits included “Auto Mode disabled by user” (0.3 per participant per week), “Timeout from Safe Basal—No Calibration” (0.3 per participant per week), and “Timeout from Safe Basal—Sensor Expired” (0.2 per participant per week). There was an average of 1.6 ± 0.9 and 1.1 ± 0.7 exits per participant per week, for the adolescent and adult groups, respectively. Approximately 25% of exits occurred overnight.

Table 2 gives the A1C and percentage of time spent across glucose ranges during the run-in period and study phase for the 24-h day, daytime, and nighttime periods, as well as the percentage of time spent in closed loop for each group in each period. The difference in overall-day TIR was 5.7% (82.3 min/day) for the overall group (*P* < 0.001, Wilcoxon signed-rank test), 10.4% (147.6 min/day) for the adolescents (*P* < 0.001), and 4.2% (60.7 min/day) for adults (*P* < 0.001) (Table 2). The daytime TIR was also statistically increased for all groups, when compared with the run-in period (Table 2).

**Table 2.** Glycemic Outcomes During the Run-in Period and Study Phase

	Overall (*n* = 157)			Adolescents (*n* = 39)			Adults (*n* = 118)		
	Run-in^a^	Study^b^	*P*	Run-in^a^	Study^b^	*P*	Run-in^a^	Study^b^	*P*
A1C, %^c^	7.5 ± 0.8	7.0 ± 0.5	<0.001^d^	7.6 ± 0.8	7.1 ± 0.6	<0.001^d^	7.5 ± 0.9	7.0 ± 0.5	<0.001^d^
24-h day									
Time in closed loop, %	—	94.9 ± 5.4	—	—	93.8 ± 5.7	—	—	95.2 ± 5.2	—
TBR <50 mg/dL	0.5 ± 0.7	0.3 ± 0.4	0.003^d^	0.6 ± 0.7	0.4 ± 0.5	0.252^d^	0.5 ± 0.7	0.3 ± 0.4	0.006^d^
TBR <54 mg/dL	0.8 ± 1.1	0.5 ± 0.6	0.001^d^	0.9 ± 1.0	0.6 ± 0.6	0.106^d^	0.8 ± 1.1	0.5 ± 0.6	0.005^d^
TBR <70 mg/dL	3.3 ± 2.9	2.3 ± 1.7	<0.001^d^	3.3 ± 2.7	2.4 ± 1.8	0.021	3.4 ± 3.0	2.3 ± 1.7	<0.001^d^
TIR 70–180 mg/dL	68.8 ± 10.5	74.5 ± 6.9	<0.001^d^	62.4 ± 9.9	72.7 ± 5.6	<0.001	70.9 ± 9.8	75.1 ± 7.3	<0.001
TAR >180 mg/dL	27.9 ± 11.0	23.1 ± 7.2	<0.001^d^	34.3 ± 10.7	24.9 ± 5.7	<0.001	25.7 ± 10.2	22.6 ± 7.5	<0.001
TAR >250 mg/dL	6.2 ± 4.7	4.6 ± 3.0	<0.001^d^	9.1 ± 5.4	5.6 ± 2.7	<0.001	5.3 ± 4.1	4.3 ± 3.0	<0.001^d^
TAR >300 mg/dL	1.7 ± 1.9	1.2 ± 1.1	<0.001^d^	2.6 ± 2.4	1.5 ± 1.1	<0.001^d^	1.4 ± 1.5	1.1 ± 1.1	0.047^d^
Daytime (6 AM–12 AM)									
Time in closed loop, %	—	94.8 ± 5.4	—	—	93.8 ± 5.9	—	—	95.2 ± 5.2	—
TBR <50 mg/dL	0.5 ± 0.7	0.3 ± 0.5	0.042^d^	0.5 ± 0.7	0.4 ± 0.5	0.342^d^	0.5 ± 0.7	0.3 ± 0.5	0.073^d^
TBR <54 mg/dL	0.8 ± 1.0	0.5 ± 0.6	0.007^d^	0.8 ± 1.0	0.6 ± 0.7	0.175^d^	0.8 ± 1.1	0.5 ± 0.6	0.020^d^
TBR <70 mg/dL	3.4 ± 3.0	2.4 ± 1.9	<0.001^d^	3.2 ± 2.7	2.5 ± 1.8	0.041	3.4 ± 3.1	2.4 ± 1.9	<0.001^d^
TIR 70–180 mg/dL	68.0 ± 10.8	72.1 ± 7.7	<0.001^d^	60.9 ± 10.4	69.8 ± 6.3	<0.001^d^	70.3 ± 9.9	72.9 ± 8.0	<0.001
TAR >180 mg/dL	28.6 ± 11.5	25.4 ± 8.1	<0.001^d^	35.8 ± 11.5	27.7 ± 6.8	<0.001	26.2 ± 10.5	24.6 ± 8.4	0.006
TAR >250 mg/dL	6.6 ± 5.2	5.3 ± 3.6	<0.001^d^	10.0 ± 6.0	6.4 ± 3.3	<0.001^d^	5.5 ± 4.4	4.9 ± 3.6	0.073^d^
TAR >300 mg/dL	1.8 ± 2.1	1.4 ± 1.4	0.002^d^	2.9 ± 2.7	1.7 ± 1.3	<0.001^d^	1.5 ± 1.8	1.3 ± 1.4	0.242^d^
Nighttime (12 AM–6 AM)									
Time in closed loop, %	—	94.9 ± 5.4	—	—	94.1 ± 5.5	—	—	95.1 ± 5.4	—
TBR <50 mg/dL	0.6 ± 1.0	0.3 ± 0.5	0.017^d^	0.7 ± 0.9	0.4 ± 0.6	0.195^d^	0.5 ± 1.0	0.3 ± 0.4	0.050^d^
TBR <54 mg/dL	0.9 ± 1.4	0.5 ± 0.7	0.006^d^	1.0 ± 1.4	0.6 ± 0.8	0.194^d^	0.8 ± 1.4	0.5 ± 0.6	0.017^d^
TBR <70 mg/dL	3.2 ± 3.5	2.0 ± 2.0	<0.001^d^	3.4 ± 3.7	2.2 ± 2.3	0.032	3.2 ± 3.5	2.0 ± 1.8	<0.001^d^
TIR 70–180 mg/dL	71.2 ± 13.6	81.5 ± 9.5	<0.001^d^	66.8 ± 12.8	81.1 ± 9.1	<0.001	72.6 ± 13.6	81.7 ± 9.7	<0.001
TAR >180 mg/dL	25.6 ± 13.6	16.4 ± 9.2	<0.001^d^	29.9 ± 13.0	16.7 ± 8.4	<0.001	24.2 ± 13.6	16.4 ± 9.5	<0.001
TAR >250 mg/dL	5.1 ± 5.1	2.6 ± 2.6	<0.001^d^	6.5 ± 5.4	3.2 ± 2.9	<0.001^d^	4.7 ± 4.9	2.5 ± 2.4	<0.001^d^
TAR >300 mg/dL	1.2 ± 2.0	0.7 ± 0.8	0.003^d^	1.7 ± 2.6	0.8 ± 1.0	0.114^d^	1.1 ± 1.7	0.6 ± 0.8	0.012^d^

All values are shown as mean ± SD.

Run-in CGM was ∼14 days and study phase was ∼90 days.

During baseline run-in period, Auto Correction bolus was inadvertently delivered on six systems.

^a^ Sensor-augmented/integrated pump, PLGM, or Auto Basal use.

^b^ Auto Basal+Auto Correction use.

^c^ Number of participants whose data comprised A1C analysis: *N* = 155 for overall, *n* = 38 for adolescents, and *n* = 117 for adults.

^d^ Wilcoxon signed-rank test.

CGM, continuous glucose monitoring; PLGM, predictive low glucose management; TAR, time spent above target glucose range; TBR, time spent below target glucose range; TIR, time spent in target glucose range.

However, a greater time spent in target range for the nighttime period was prominent, where the difference in TIR was 10.3% for the overall group (*P* < 0.001, Wilcoxon signed-rank test), 14.3% for adolescents (*P* < 0.001), and 9.1% for adults (*P* < 0.001). The overall group 24-h day TBR reduced by 1.0% (14.4 min) and TAR reduced by 4.7% (67.9 min). Although there were reductions in both metrics for the overall group daytime and nighttime periods, the TBR and TAR for all time periods were reduced or unchanged for adolescents and adults (Table 2).

**FIG. 3.**
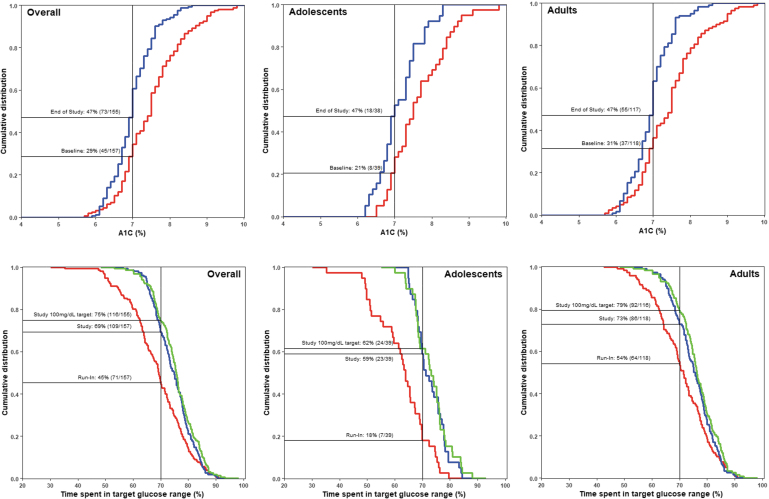
Cumulative distributions of participants achieving A1C <7.0% and TIR >70%. The proportion of overall, adolescent, and adult participants with A1C of <7.0% (top panel) and time spent in target glucose range (TIR) of >70% (bottom panel) are shown for the baseline/run-in period (red), the overall study phase when both the 100 and 120 mg/dL targets were set and factored (blue) and when only the 100 mg/dL glucose target was set (green). System use during the run-in period included SAP, PLGM, or Auto Basal therapy, whereas that for the study phase included Auto Basal and Auto Correction. During baseline run-in, Auto Correction bolus was inadvertently delivered on six systems.

During half of the study phase, a glucose target of either 100 or 120 mg/dL was programmed. A comparison of the proportion of participants achieving a recommended A1C of <7% at baseline versus the end of study (when both the 100 and 120 mg/dL target setting were factored) is shown for all groups (Fig. 3). The leftward shifts in cumulative distribution curves from baseline to end of study, which are observed in all groups, indicate a greater percentage of participants achieving an A1C of <7%. Figure 3 also shows the reverse cumulative distribution for participants achieving the recommended TIR of >70% during the run-in period and during the study phase for both target settings (in addition to the 100 mg/dL target setting).

For the TIR of >70%, rightward shifts in the distributions for the 100 mg/dL target and the 100 + 120 mg/dL target curves were observed from that of the run-in period, indicating a greater percentage of participants achieving the TIR goal. This was observed in all groups. For the overall group, it was an increase from 45% (*n* = 71/157) to 69% (*n* = 109/157) for the overall study phase (both targets factored together) and to 75% (*n* = 116/155) at the 100 mg/dL target. In adolescents, the proportion increased from 18% (*n* = 7/39) to 59% (*n* = 23/39) for both targets, and to 62% (*n* = 24/39) at the 100 mg/dL glucose target. Similarly, and in the adults, it increased from 54% (64/118) to 73% (86/118) for both targets, and to 79% (92/116) at the 100 mg/dL glucose target.

The composite of the percentage of overall participants achieving a recommended TIR of >70%, TBR <70 mg/dL of <4%, and TBR <54 mg/dL of <1% during run-in period was 31%. This increased to 60% for both targets and the 100 mg/dL target setting. In adolescents, it increased from 15% to 51% for both targets, whereas, in adults, it increased from 36% to 63%.

An exploratory subgroup analysis was performed for TIR and TBR <70 mg/dL based on participants with different AIT settings. At the 100 mg/dL target, and when the AIT setting was set at >4 h (*n* = 4 total, *n* = 0 adolescents) versus 2 h (*n* = 29 total, *n* = 7 adolescents), TIR increased from 70.6% to 78.8% (Spearman correlation, *R* = −0.25, [−0.38, −0.1]). The TBR <70 mg/dL reduced from 4.8% to 2.6% (*R* = −0.04 [−0.11, 0.19]). While at the 120 mg/dL target and when AIT was set at >4 h (*n* = 2 total, *n* = 0 adolescents) versus 2 h (*n* = 26 total, *n* = 6 adolescents), TIR increased from 68.1% to 75.0% (*n* = 26 total, *n* = 6 adolescents) (*R* = −0.16 [−0.30, −0.01]), although the TBR <70 mg/dL increased from 1.4% to 1.9% (*R* = −0.03 [−0.18, 0.11]).

### SG, glucose variability, and insulin delivered

Table 3 lists the SG, CV of SG, and insulin delivered during the 24-h, daytime, and nighttime periods of the run-in period and study phase. From run-in period to end of study, the 24-h day mean SG and glucose variability (CV of SG) were reduced for the overall group and adults. For the daytime period, however, overall group CV of SG (although 34.5% in adults and 36.7% in adolescents, at study start) did not change.

**Table 3.** Sensor Glucose, Glucose Variability, and Insulin Delivery, During the Run-in Period and Study Phase

	Overall (*n* = 157)			Adolescents (*n* = 39)			Adults (*n* = 118)		
	Run-in^a^	Study^b^	*P*	Run-in^a^	Study^b^	*P*	Run-in^a^	Study^b^	*P*
24-h day									
SG, mg/dL	153 ± 16	148 ± 10	<0.001^d^	162 ± 16	150 ± 8	<0.001	151 ± 15	147 ± 11	<0.001
CV of SG, %	35.0 ± 5.0	34.2 ± 4.1	0.003	36.5 ± 4.8	35.7 ± 4.1	0.202	34.5 ± 5.0	33.7 ± 3.9	0.008
Total insulin, units	54.9 ± 25.8	56.7 ± 28.4	0.002^d^	60.0 ± 16.3	63.1 ± 17.3	0.007	53.2 ± 28.1	54.6 ± 31.0	0.103^d^
Total basal insulin, units	25.1 ± 13.6	23.6 ± 13.2	<0.001^d^	25.2 ± 8.7	24.4 ± 8.4	0.265	25.1 ± 14.9	23.3 ± 14.5	<0.001^d^
Total bolus insulin, units	29.8 ± 14.8	33.1 ± 16.8	<0.001^d^	34.8 ± 10.9	38.7 ± 10.1	0.002	28.1 ± 15.5	31.3 ± 18.2	<0.001^d^
Auto Correction, units	0.0 ± 0.4	7.5 ± 6.1	<0.001^d^	0.0 ± 0.2	8.3 ± 3.3	<0.001	0.0 ± 0.5	7.3 ± 6.7	<0.001^d^
Auto Correction, %^c^	0.2 ± 1.4	22.0 ± 9.3	<0.001^d^	0.1 ± 0.5	21.6 ± 7.0	<0.001	0.2 ± 1.6	22.1 ± 10.0	<0.001^d^
Daytime (6 AM–12 AM)									
SG, mg/dL	155 ± 17	151 ± 12	<0.001^d^	164 ± 17	154 ± 10	<0.001^d^	151 ± 16	150 ± 12	0.113
CV of SG, %	35.0 ± 5.1	34.5 ± 4.0	0.157^d^	36.7 ± 4.9	36.0 ± 4.1	0.261	34.5 ± 5.1	34.0 ± 3.8	0.129
Total insulin, units	46.4 ± 21.7	47.5 ± 23.6	0.024^d^	51.4 ± 13.7	53.4 ± 14.2	0.061	44.7 ± 23.6	45.6 ± 25.7	0.250^d^
Total basal insulin, units	18.7 ± 10.1	17.2 ± 9.8	<0.001^d^	18.6 ± 6.6	17.7 ± 6.1	0.092	18.7 ± 11.0	17.0 ± 10.8	<0.001^d^
Total bolus insulin, units	27.6 ± 13.8	30.3 ± 15.3	<0.001^d^	32.7 ± 10.1	35.7 ± 9.3	0.017	26.0 ± 14.5	28.5 ± 16.5	<0.001^d^
Auto Correction, units	0.0 ± 0.4	5.9 ± 4.8	<0.001^d^	0.0 ± 0.2	6.5 ± 2.7	<0.001^d^	0.0 ± 0.4	5.7 ± 5.4	<0.001^d^
Auto Correction, %^c^	0.2 ± 1.3	18.8 ± 8.7	<0.001^d^	0.1 ± 0.5	18.5 ± 6.6	<0.001^d^	0.2 ± 1.5	18.9 ± 9.2	<0.001^d^
Nighttime (12 AM–6 AM)									
SG, mg/dL	150 ± 19	140 ± 13	<0.001	156 ± 19	140 ± 12	<0.001	148 ± 19	140 ± 14	<0.001
CV of SG, %	33.1 ± 6.1	30.6 ± 5.2	<0.001^d^	34.1 ± 5.9	32.0 ± 5.0	0.008	32.7 ± 6.1	30.1 ± 5.2	<0.001^d^
Total insulin, units	8.6 ± 4.8	9.3 ± 5.5	<0.001^d^	8.7 ± 3.5	9.8 ± 3.8	<0.001^d^	8.5 ± 5.1	9.1 ± 5.9	<0.001^d^
Total basal insulin, units	6.5 ± 3.7	6.4 ± 3.7	0.990^d^	6.6 ± 2.3	6.7 ± 2.3	0.593	6.4 ± 4.0	6.4 ± 4.0	0.672^d^
Total bolus insulin, units	2.1 ± 2.1	2.8 ± 2.5	<0.001^d^	2.1 ± 2.1	3.1 ± 2.2	<0.001	2.1 ± 2.0	2.8 ± 2.6	<0.001^d^
Auto Correction, units	0.0 ± 0.1	1.7 ± 1.5	<0.001^d^	0.0 ± 0.0	1.8 ± 0.9	<0.001^d^	0.0 ± 0.1	1.6 ± 1.6	<0.001^d^
Auto Correction, %^c^	0.5 ± 3.6	66.1 ± 22.4	<0.001^d^	0.3 ± 1.3	69.9 ± 20.6	<0.001^d^	0.5 ± 4.1	64.9 ± 22.9	<0.001^d^

All values are shown as mean ± SD.

Run-in CGM was ∼14 days and study phase was ∼90 days.

During baseline run-in period, Auto Correction bolus was inadvertently delivered on six systems.

^a^ Sensor-augmented/integrated pump, PLGM, or Auto Basal use.

^b^ Auto Basal+Auto Correction use.

^c^ Factored as a percentage of total bolus insulin.

^d^ Wilcoxon signed-rank test.

CV, coefficient of variation; SG, sensor glucose.

**FIG. 4.**
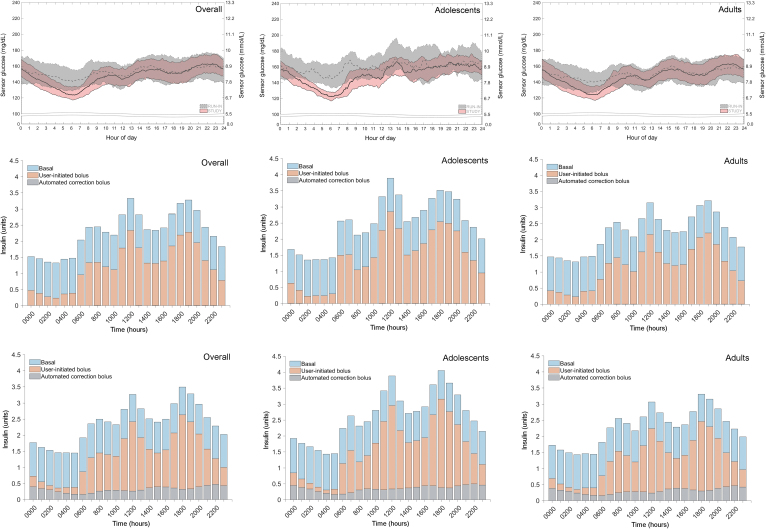
SG and insulin delivered profiles, during the run-in period and study phase. The median and IQRs of SG levels (top panel) and the units of insulin delivered during the run-in period (middle panel) and study phase (bottom panel), across the 24-h day for the overall, adolescent (14–21 years), and adult (>21–75 years) groups are shown. For SG, medians (solid), and IQR intervals (dotted) of the run-in period (gray) and study phase (pink) appeared to vary most during the nighttime period (12 AM–6 AM). For insulin delivered, the units of total basal (blue), user-initiated bolus (orange), and automated correction bolus (gray) are shown. The study phase Auto Correction bolus averaged 20% of total bolus for all groups. System use during the run-in period included SAP, PLGM, or Auto Basal therapy, whereas that for the study phase included Auto Basal and Auto Correction. During baseline run-in period, Auto Correction bolus was inadvertently delivered on six systems. IQRs, interquartile ranges; SG, sensor glucose.

The changes in SG and glucose variability for the remaining nighttime period, which were reduced during the AHCL study phase for all groups, were evidenced by the lowered medians and narrowed interquartile ranges shown in the SG profiles (Fig. 4). Relating to this, the mean total of insulin delivered across the 24-h day and nighttime periods for all three groups was increased and was primarily due to an increase in total bolus insulin delivery (Table 3).

The amount of Auto Correction insulin, as a percentage of total bolus insulin, during the 24-h day and daytime periods was similar across the groups: 22.0% and 18.8% for the overall group, respectively; 21.6% and 18.5% for adolescents, respectively; and 22.1% and 18.9%, for adults, respectively. During the nighttime (i.e., sleeping hours), most of the total bolus insulin delivered for the overall, adolescent, and adult groups was through Auto Correction, where the percentage reached 66.1%, 69.9%, and 64.9% respectively. The changes in total insulin and total bolus insulin delivered from run-in period to study phase were greater for the adolescents than for the adults. Although total basal insulin was reduced in the overall group and the adults, this was only observed for the 24-h day and daytime periods.

## Discussion

This 3-month pivotal trial with a total 14,134 days of AHCL Auto Basal and Auto Correction use had no device-related SAEs and no serious or unanticipated device-related effects. There were also no episodes of severe hypoglycemia or DKA during the Auto Basal and Auto Correction-enabled study phase. This safety profile is similar to that observed in the 3-month MiniMed 670G system pivotal trials conducted in children,^16^ adolescents, and adults,^17^ as well as the current AHCL system when investigated in the short randomized and controlled studies.^26,28,31,34^

Glycemic outcomes of this study demonstrated reduced A1C and increased overall (24-h day) TIR in adolescents and adults using the AHCL system, when compared with a run-in period of SAP, PLGMs or automated basal insulin delivery use. A further increased TIR (>81%) was observed during the nighttime. There were also reductions in overall, daytime, and nighttime TBR <70 mg/dL and TAR >180 mg/dL for all groups. An important note is that the reduction in TAR >180 mg/dL was greatest in the adolescent group, regardless of the period of day, and ranged from −8.1% to −13.1%. This achievement met the international recommended goal of <25% for TAR >180 mg/dL^15^ and is substantial given the potential risk of diabetes complications development in youth and adolescents.^35^

The glycemic improvements reported in RCTs assessing AHCL system use for ≥4 weeks support current study findings of glycemic benefits. A 4-week cross-over RCT in children, adolescents, and adults (*n* = 59 aged 23.3 ± 14.4 years) demonstrated an overall 96.4% of time in closed loop, and a significantly reduced SG (across all age groups) compared with baseline, and PLGM therapy control.^31^ Furthermore, with AHCL use, TIR significantly increased by 11.8 ± 7.4% in the 7–13 years cohort, 14.4% ± 8.4% in the 14–21 years cohort, and by 11.9 ± 9.5% in the adults compared with PLGM therapy.

In a previous study, overall group TIR was improved (i.e., optimized) when the system was programmed at the lower 100 mg/dL versus the 120 mg/dL glucose target (*n* = 51 vs. *n* = 23, 72% vs. 65%, respectively).^31^ Optimization was also observed in this study. Although Collyns et al. did not analyze AIT setting, a recent short longitudinal MiniMed 780G system study (*n* = 52 adolescents and adults, aged 43 ± 12 years) showed that at the most aggressive system settings consisting of a 100 mg/dL glucose target and AIT of 2 h, there was an overall 97% of time spent in closed loop, a TIR that increased from 67.3% to 79.6%, and a TAR >180 mg/dL that reduced from 29.4% to 17.3%, without change in TBR <70 mg/dL or TBR <54 mg/dL.^32^

The FLAIR RCT was the first study to compare 12-week use of both the MiniMed 670G system versus AHCL system in adolescents and young adults (*n* = 113, mean of 19 ± 4.0 years of age) and it demonstrated A1C that improved from 7.9% at baseline run-in period, to 7.6% with HCL, and to 7.4% with AHCL.^30^ The 24-h day TIR improved from 57% to 63% and 67%, respectively; the difference in overall TIR with AHCL versus HCL use was significant. Similar to this study, improved TIR was observed for the study's daytime (64% with AHCL vs. 61% with HCL) and nighttime (74% with AHCL vs. 70% with HCL) periods. Time spent in hyperglycemia (>180 mg/dL) for the daytime period, a coprimary outcome in the RCT, was statistically reduced with AHCL versus HCL use (34% vs. 37%, respectively). Although the FLAIR study reported one severe hypoglycemic event during AHCL use, an exploratory analysis on the proportion of participants achieving TIR at >70% and TBR <54 mg/dL at <1% demonstrated that 21% using the MiniMed 670G system and 30% using the AHCL system reached that combined outcome. Present study findings were similar, where the percentage of adolescents achieving the combined recommended time in target range and below range (both TBR <70 mg/dL at <4% and TBR <54 mg/dL at <1%) increased from 15% to 51%.

A greater percentage of adults achieved the aforementioned targets after AHCL use, as well. Although both this study and the FLAIR trial demonstrated clear trends in achieved recommended goals, the increased percentage reaching these targets in the former may be due to fewer adolescents (*n* = 22/39) [56%] using HCL during the run-in period and only 6/112 [5%] in the FLAIR trial having the AIT setting of 2–2.5 h.

Similar to both the Collyns et al. and FLAIR RCTs, improvements in glycemia during the AHCL-enabled study phase overnight period were greater than the 24-h day or daytime period. This was evident in both the adolescent and adult groups who had relatively well-controlled A1C and SG at study start. Although improvement in daytime CV of SG was not observed for either group, their baseline CV of SG approximated the 36% level considered “stable” for individuals with T1D,^36,37^ and the Auto Correction function appeared to reduce daytime hyperglycemia.

Reduced exposure to hyperglycemia is very important for youth with T1D who often have difficulty managing hyperglycemia throughout any period of the day. With algorithm-driven insulin bolus corrections, adolescents have been observed to show remarkable improvements in overall TIR and/or SG of >10% for the former and >12 mg/dL for the latter, compared with control.^26,31^ Improved TIR and SG have also been observed in adolescents (*n* = 31, aged <18 years, of 112 total participants) during the 6-month randomized trial of the t:slim X2 with Control-IQ technology system that also has both automated basal and bolus correction functions, where TIR increased from 61% to 71% and SG decreased from 166 to 156 mg/dL.^8^

More recently, and in an even younger cohort (*n* = 112, aged 6–13.9 years), the 3-month pivotal trial of the tubeless OmniPod™ 5 automated insulin delivery system (Insulet Corporation, Acton, MA) has demonstrated a TIR that increased from 52.5% to 68% and SG that decreased from 183 to 160 mg/dL.^38^ All of these findings are of substantial import in youth with T1D, as TIR has been shown to associate with A1C^39,40^ and is evolving to become a standard predictor for diabetes complications risk.^41–43^

The impact of this study's AHCL algorithm on insulin delivery, and its role in the reduction of SG, especially during the nighttime, was notable. For instance, overall user-initiated insulin boluses across the 24-h period (either due to corrective insulin or meal announcement) averaged 99.8% of the total bolus insulin delivered, during the run-in period. During AHCL use, however, user-initiated insulin delivery averaged 78% of the total bolus insulin delivered, with overall Auto Correction comprising 22% of total bolus insulin, similar as that seen in Collyns et al.^31^

These characteristics of algorithm- versus user-initiated insulin delivery between the run-in period and study phase show that the AHCL system provided automation that reduced hyperglycemic excursion without compromising the recommended target for TIR of >70% and TBR of <4%, while reducing insulin dosing burden.

The strengths of this study are the inclusion of both adolescents and adults, multiple investigation centers, and 14,134 days of closed-loop system use without an episode of severe hypoglycemia or DKA. The limitations and weaknesses of this study are the nonrandomized design that did not involve a control group. The different glucose target settings for half of the study phase, small sample size for each age cohort, and the inclusion of individuals with relatively targeted baseline glycemia (7.5% ± 0.8% A1C) and >6 months of prior pump experience limit generalizability to a larger or more diverse population.

However, the reported findings from previous short or longer duration RCTs assessing AHCL use^26,28,30,31^ are rather comparable with the present study results, all of which demonstrate that the system is safe and provides significant improvement in most glycemic parameters across a broad age range of individuals with T1D. Future longer term and real-world studies may better determine how well AHCL therapies improve diabetes management in larger groups and reduce the risk of diabetes complications.

## Conclusion

The MiniMed AHCL pivotal trial in adolescents and adults with T1D demonstrates that the system is safe and allows more individuals to reach internationally recommended glycemic targets. The recent Conformité Européenne mark of the MiniMed 780G system for individuals with diabetes at least 7 years of age, will enable longer term analyses of glycemic targets achieved with this advanced automated insulin delivery therapy.

## MiniMed AHCL System Study Group

B.W.B. (Atlanta Diabetes Associates, Atlanta, Georgia), R.L.B. (Rainier Clinical Research Center, Renton, Washington), A.L.C. (International Diabetes Center, HealthPartners Institute, Minneapolis, Minnesota), X.C. (Medtronic), M.C. (Diablo Clinical Research Center, Walnut Creek, California), T.L.C. (Medtronic), S.K.G. (Barbara Davis Center of Childhood Diabetes, Aurora, Colorado), B.G. (Medtronic), R.A.M.J. (Medtronic), K.B.K. (SoCal Diabetes, Torrance, California), M.S.K. (Diabetes & Glandular Disease Clinic, San Antonio, Texas), D.R.L. (Rocky Mountain Diabetes and Osteoporosis Center, Idaho Falls, Idaho), Louis J. Lintereur (Medtronic), Margaret Liu (Medtronic), Neha Parikh (Medtronic), Fen Peng (Medtronic), A.P.-T. (Scripps Whittier Diabetes Institute, La Jolla, California), R.P.-B. (University of Michigan, Division of Metabolism, Endocrinology and Diabetes, Ann Arbor, Michigan), J.H.C.R. (Endocrine Research Solutions, Inc., Roswell, Georgia), A.S.R. (Medtronic), A.R. (Medtronic), J.L.S. (Yale University School of Medicine Pediatric Endocrinology, New Haven, Connecticut), J.S. (Medtronic), D.I.S. (University of South Florida Diabetes & Endocrinology, Tampa, Florida), Kamalpreet Singh, MD (Texas Diabetes & Endocrinology, Round Rock, Texas), R.H.S. (Barbara Davis Center of Childhood Diabetes, Aurora, Colorado), J.R.T. (Arkansas Diabetes and Endocrinology Center, Little Rock, Arkansas), M.V. (Medtronic), R.A.V. (Medtronic) and Di Wu (Medtronic).
